# Accuracy of half-guided implant placement with machine-driven or manual insertion: a prospective, randomized clinical study

**DOI:** 10.1007/s00784-021-04087-0

**Published:** 2021-08-16

**Authors:** Kristof Orban, Endre Varga, Peter Windisch, Gabor Braunitzer, Balint Molnar

**Affiliations:** 1 Faculty of Dentistry, Department of Periodontology, Semmelweis University, Budapest, Hungary; 2 dicomLAB Dental, Ltd., Szeged, Hungary

**Keywords:** Dental implants, Guided surgery, Intraoral digital scan, Accuracy, Machine-driven implant insertion, Manual implant insertion

## Abstract

**Objectives:**

To compare the accuracy of implant placement performed with either a surgical motor or a torque wrench as part of a half-guided surgical protocol.

**Materials and methods:**

Implant insertion with half-guided surgical protocol was utilized by surgical motor (machine-driven group) or torque wrench (manual group) in the posterior maxilla. After the healing period, accuracy comparison between planned and actual implant positions was performed based on preoperative cone beam computed tomography and postoperative digital intraoral scans. Coronal, apical, and angular deviations, insertion time, and insertion torque were evaluated.

**Results:**

Forty patients were treated with 1 implant each; 20 implants were inserted with a surgical motor and 20 implants with a torque wrench. Global coronal and apical deviations were 1.20 ± 0.46 mm and 1.45 ± 0.79 mm in the machine-driven group, and 1.13 ± 0.38 mm and 1.18 ± 0.28 mm in the manual group (respectively). The mean angular deviation was 4.82 ± 2.07° in the machine-driven group and 4.11 ± 1.63° in the manual group. Mean insertion torque was 21.75 ± 9.75 Ncm in the machine-driven group, compared to 18.75 ± 7.05 Ncm in the manual group. Implant placement duration was 9.25 ± 1.86 s in the machine-driven group at a speed of 50 rpm, and 36.40 ± 8.15 s in the manual group.

**Conclusion:**

No significant difference was found between the two groups in terms of accuracy and mean insertion torque, while machine-driven implant placement was significantly less time-consuming.

**Clinical relevance:**

Optimal implant placement accuracy utilized by half-guided surgical protocol can be achieved with both machine-driven and torque wrench insertion.

**Trial registration:**

ID: NCT04854239

## Introduction

Dental implant therapy is a widespread, safe, and predictable treatment option to replace missing teeth. Long-term success is determined, among other factors, by the amount of alveolar bone, the condition of the surrounding soft tissues, and the accuracy of implant placement, as well as the accuracy of the implant-borne restorations.

Conventional implant position planning is based on the shape and volume of native alveolar bone as determined in two-dimensional radiographs. Using cone beam computed tomography (CBCT) scans, the three-dimensional structure of the alveolar bone can be precisely mapped, which allows optimal implant positioning both prosthetically and by minimizing the risk of damage to neighboring anatomical structures such as the mandibular nerve or the sinuses [1]. In the case of single-tooth replacement, the axis and distance of neighboring teeth may help the clinician to insert the implant in a prosthetically favorable position. Larger edentulous sites make implant placement more difficult and a surgically driven approach without recognizable anatomical landmarks often results in prosthetically inappropriate implant position and angulation. In turn, this may lead to an esthetically and functionally suboptimal outcome [2–5].

Due to the increased patient demand for aesthetic implant-borne restorations that resemble the lost natural dentition as much as possible, implant positioning accuracy has gradually become a central issue in implant dentistry. To achieve optimal aesthetics and cleansability, a prosthetically driven surgical approach was reported to increase implant placement precision, which allows for easily retrievable screw-retained restorations, eliminating the risk of harmful submucosal cement residues [6]. The introduction of computer-aided design (CAD) for guided implant surgery meant the beginning of a new era in implant dentistry [7]. With the help of CAD software, it is possible for the clinician to first determine the ideal restoration design and then to plan the position of the implants in a way that enables the realization of the desired prosthetic outcome. A prerequisite for optimal virtual implant positioning and execution of guided surgery is the 3D reconstruction of peri-implant hard tissues either simultaneously or in a staged approach. Frequently, the condition of hard tissues is compromised at the planned implant position, thus hard and soft tissue augmentation procedures may be required before or at the time of implant placement.

Several methods have been described in the literature to increase the accuracy of implant positioning. Studies have confirmed the superiority of guided implant placement over freehand implant placement [8]. A diagnostic wax-up-based surgical template fabricated by the dental technician is a conventional but efficient and cost-effective solution. Nevertheless, this method only allows for more precise, although freehand implant positioning without vertical depth control, strongly depending on the individual stent design. Computer-assisted implant planning and template-guided implant placement represent a more advanced treatment modality. The alignment of a CBCT dataset and the digital image of a diagnostic wax-up in a CAD planning software allows the clinician to virtually plan the implant position in three dimensions. This approach makes it possible to plan directly with the digital model of the implant to be inserted and to determine its exact angulation and position within the bone, which increases the accuracy of planning to a considerable extent [9, 10]. Subsequently, the digital plan is converted into an individually fabricated stereolithographic surgical template.

Such templates are used according to different protocols defined by the degree of guidance. The pilot protocol uses the template only for the initial drill (“pilot drill”), which guides subsequent osteotomies and implant placement. The half- (or partially) guided protocol uses the template for all osteotomies; only implant placement is performed without the template. Finally, the fully guided protocol uses the template during the complete drilling sequence as well as for implant placement. A major advantage of the guided approach is that it greatly reduces the role of the surgical skills. Operator experience has no significant effect on the outcome—if the outcome is inaccurate, it is mostly because of the malpositioning of the surgical guide [11, 12].

In contrast, Joda et al. in 2018 in their consensus report summarized that static computer-aided implant surgery, in terms of postoperative pain, discomfort, and intraoperative complications, is not proven superior to conventional implant surgery [13]. Error during conventional or digital impression, implant malpositioning during digital planning, or surgical guide inaccuracy can influence final implant position. If the positioning of the surgical guide is accurate, the half-guided surgical protocol cannot completely eliminate implant placement inaccuracy, given that the final step, insertion, is unguided. There is no clear recommendation in the literature as to whether the clinician can achieve higher accuracy with a surgical motor or a torque wrench and, as a matter of fact, hardly any literature is available on this question. The only work that explicitly mentions the choice between the manual and machine-driven options leaves the decision to the clinician without discussing the possible effects on accuracy [14].

The primary aim of this study was to compare the accuracy of implant placement performed with either a surgical motor or a torque wrench as part of a half-guided surgical protocol. Secondary analyses were conducted regarding the duration of implant insertion and maximum insertion torque.

## Materials and methods

### Patient demographics and allocation

Forty patients (21 women and 19 men, mean age: 49 ± 10 years) were selected and treated at the Department of Periodontology, Semmelweis University, Budapest, Hungary, between January 2017 and January 2019. The study conformed to the tenets of the Declaration of Helsinki (as amended in 2013) in all respects. The study protocol was approved by the Regional and Institutional Committee of Science and Research Ethics at the Semmelweis University (Approval Number: SE TUKEB 7/2017). Surgical interventions were undertaken with the understanding and written consent of each subject.

The inclusion criteria were as follows: at least one edentulous maxillary premolar or molar site treated successfully by sinus floor elevation with a xenogenic bone substitute (cerabone, botiss biomaterials, Zossen, Germany) confirmed by preoperative CBCT. Full-mouth plaque and bleeding scores (FMPS and FMBS) < 20%, as well as good patient compliance (including willingness to participate in the follow-up procedures). All patients had to understand the study procedure as confirmed by a signed informed consent.

The exclusion criteria were as follows: clinically relevant diseases (e.g., diabetes, rheumatism, cancer), untreated periodontitis, systemic steroid or bisphosphonate use, acute or chronic inflammatory processes. All clinical and radiographic parameters were ascertained by an experienced examiner to check the eligibility of each patient for the study.

Six months after sinus elevation, the patients were allocated to either of two groups before implant surgery with a computer-generated randomization scheme (https://www.randomizer.org). The groups were defined by the method of implant insertion. Patients in both groups received one implant each according to a half-guided protocol, but patients in the machine-driven group had their implant inserted by means of a surgical motor, while patients in the manual group had their implant inserted by means of a torque wrench.

### Preoperative imaging and planning

**Fig. 1:**
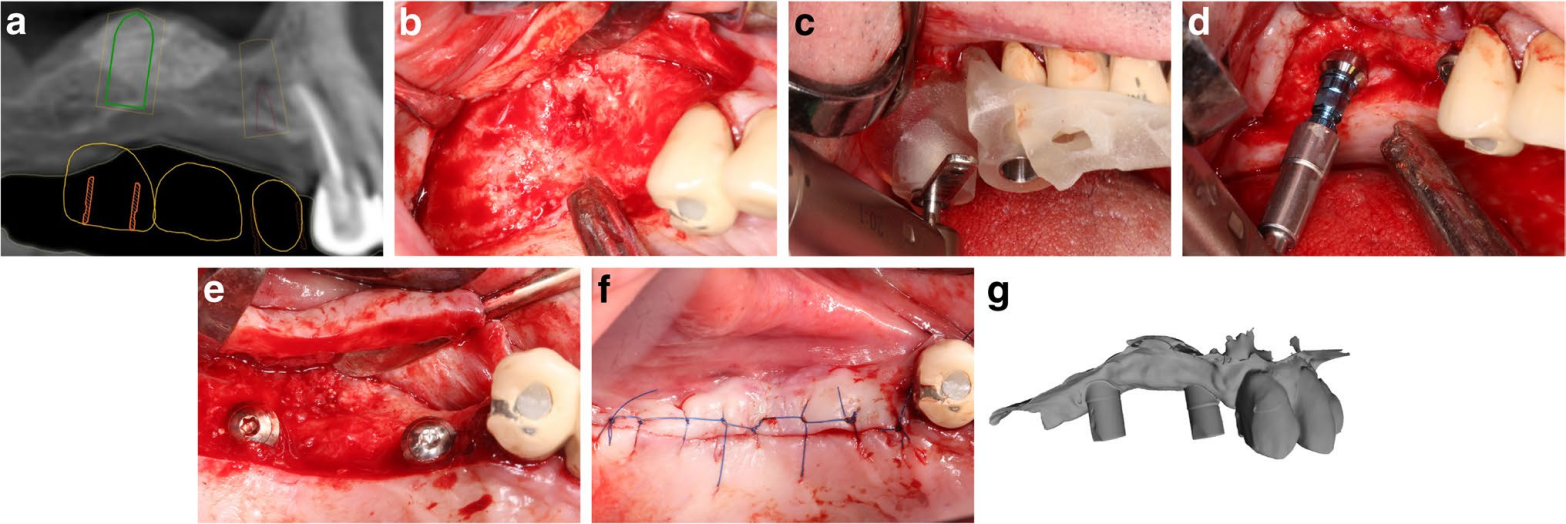
Machine-driven group (patient no. 26) **a** Planned implant position. **b** Reentry 6 months after sinus elevation. **c** Half-guided implant surgery. **d** Motor-driven implant placement. **e** Inserted implant. **f** Wound closure. **g** Intraoral scan at implant uncovery

**Fig. 2:**
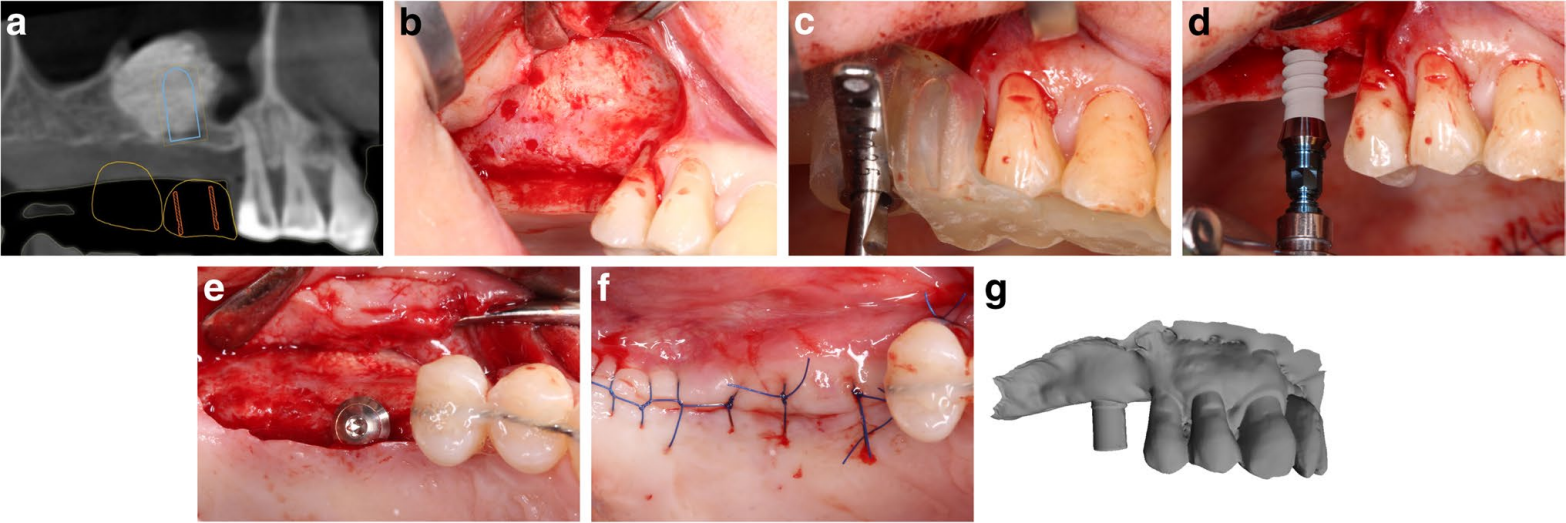
Manual group (patient no. 2). **a** Planned implant position. **b** Reentry 6 months after sinus elevation. **c** Half-guided implant surgery. **d** Manual implant placement. **e** Inserted implants. **f** Wound closure. **g** Intraoral scan at implant uncovery

Six months after sinus floor elevation, custom-made surgical guides were prepared for each patient according to the SMART Guide workflow (SMART Guide, dicomLAB Dental, Szeged, Hungary). The workflow was described in detail elsewhere [5]. Briefly, C-silicone impressions (Zetaplus, Zhermack, Badia Polesine, Italy) were taken of the patients’ upper dentition with a plastic impression tray containing radiographic markers. For the digital planning, a CBCT scan (Planmeca Viso, Planmeca, Helsinki, Finland) was taken of each patient with the impression in situ, followed by another scan of the impression alone. In this imaging protocol, the impression of the patient’s dentition serves as the model for the surgical template to be printed, and the patient’s CBCT scan is used to generate a three-dimensional model in which the position of the implants can be planned. One 4.1- × 10-mm Straumann RN Standard Plus implant (Straumann, Basel, Switzerland) was planned for each patient in the previously augmented sinus area. If necessary, further implants were planned, and length and shape of the implants were chosen based on individual patients’ needs. One implant per patient placed into the augmented sinus area was selected for the study, and the rest of the implants (if any) were excluded to standardize bone quality and implant parameters at the study sites. Following the prosthetic implant planning procedure, dentally supported stereolithographic surgical templates were fabricated (Figs. 1 and 2).

### Preoperative care

All patients underwent supra- and subgingival scaling 4 weeks before the implant surgery. The patients also received individualized oral hygiene instructions and maintained a high level of oral hygiene throughout the treatment period (FMPS, FMBS ≤ 25%). Immediately before the surgery, the patients were instructed to rinse with chlorhexidine digluconate 0.2% mouthrinse (Curasept ADS 220, Curaden AG, Kriens, Switzerland) for 2 min.

### Implant placement

Implant surgeries were performed under local anesthesia (4% articaine-hydrochloride with 0.0001% epinephrine—Ultracain DS Forte, Sanofi-Aventis, Paris, France) by the same surgeon (BM). A slightly palatal incision was placed on the keratinized mucosa of edentulous sites with No. 15 blades (Aesculap, Braun AG, Tuttlingen, Germany), continued intracrevicularly at the adjacent teeth. If deemed necessary, a single remote vertical releasing incision was placed mesially. A full-thickness mucoperiosteal buccal flap was reflected with elevators. Flap elevation was not performed on the palatal side. Implant osteotomy was performed in the pre-planned position through the custom-made surgical guide, with a surgical motor (NSK Surgic Pro, Nakanishi, Kanuma Tochigi, Japan) using a universal implant drill kit designed for guided surgery (SMART Guide Universal Kit, dicomLAB Dental, Szeged, Hungary).

In the machine-driven group, implant insertion was performed with a 20:1 surgical contra-angle handpiece. The surgical motor was configured to 50 RPM and a maximum torque of 35 Ncm without water cooling. In the manual group, implant insertion was performed with a torque wrench. Duration of implant insertion was measured in seconds from the time when implant touched the bone surface until the final position was reached. Measurements were registered by a stopwatch. After implant insertion, a depth control device was placed into the implants through the sleeves to verify the vertical position compared to the guide.

Implant closure screws were placed for submucosal healing. After implant insertion, 5–0 horizontal mattress and single interrupted sutures (Supramid, Braun AG, Tuttlingen, Germany) were placed to close the mucoperiosteal flap and to reach a tension-free wound closure. Sutures were removed 7 days postoperatively (Figs. 1 and 2).

### Implant reentry and intraoral scanning for the positional analyses

After 3 months of healing, implant reentry was performed in local anesthesia. PMMA implant scanbodies (CARES CI RD Mono Scanbody, Straumann, Basel, Switzerland) were connected to the implants, and a digital impression was taken with an intraoral scanner (Planmeca PlanScan; Planmeca, Helsinki, Finland) in the regions of interest (ROI) under partial isolation (Optragate, Ivoclar Vivadent, Schaan, Liechtenstein). At least 3 neighboring teeth were involved in each ROI. After recording the implant position, healing abutments were connected and 5–0 sutures (Supramid, Braun AG, Tuttlingen, Germany) were placed if necessary. Sutures were removed 7 days postoperatively (Figs. 1 and 2).

### Postoperative care

After implant placement, systemic antibiotic therapy (penicillin with clavulanic acid 2 × 1000 mg/day; Augmentin Duo, GlaxoSmithKline, Brentford, UK), and non-steroidal anti-inflammatory drugs (diclofenac-sodium 4 × 50 mg/day; Cataflam, Novartis International AG, Basel, Switzerland) were prescribed for 7 days in order to avoid infections and to decrease swelling and pain. In case of penicillin allergy, 4 × 300 mg/day clindamycin (Dalacin C, Pfizer, New York, USA) was administered for 7 days. For chemical plaque control, 0.2% chlorhexidine digluconate mouthrinse (Curasept ADS 220, Curaden AG, Kriens, Switzerland) was prescribed 3 times a day. After the delivery of screw-retained fixed partial dentures, the patients were enrolled in a periodontal maintenance program.

### Data analysis

The primary analyses were concerned with the positional accuracy of the inserted implants. Secondary analyses were conducted regarding the duration of implant insertion (s) and maximum insertion torque (Ncm).

Accuracy analysis was conducted in Amira 5.4.0 (Thermo Fisher Scientific, USA) with dedicated algorithms (dicomLAB Dental, Hungary). The present measurement protocol was previously published by our group [5]. Preoperative CBCT scans were aligned with postoperative intraoral scans in the coordinate system of the surgical plan. We applied this approach to minimize patients’ radiation exposure. After registering the pre- and postoperative images, the planned implant positions were extracted from the guided surgery plan and transferred to a three-dimensional digital implant model that corresponded in all its dimensions to the implant that had been inserted. Then, a digital model of the scan abutment was aligned to the actual scan abutment of the postoperative image. In this procedure, the position of the inserted implant was defined by the position of the scan abutment, as the two were directly connected and their axes fell in the same line.

Having determined the position of the inserted implant, it became possible to compare the spatial relation of the planned and actual implant positions with the help of a custom algorithm written for this purpose.

**Fig. 3:**
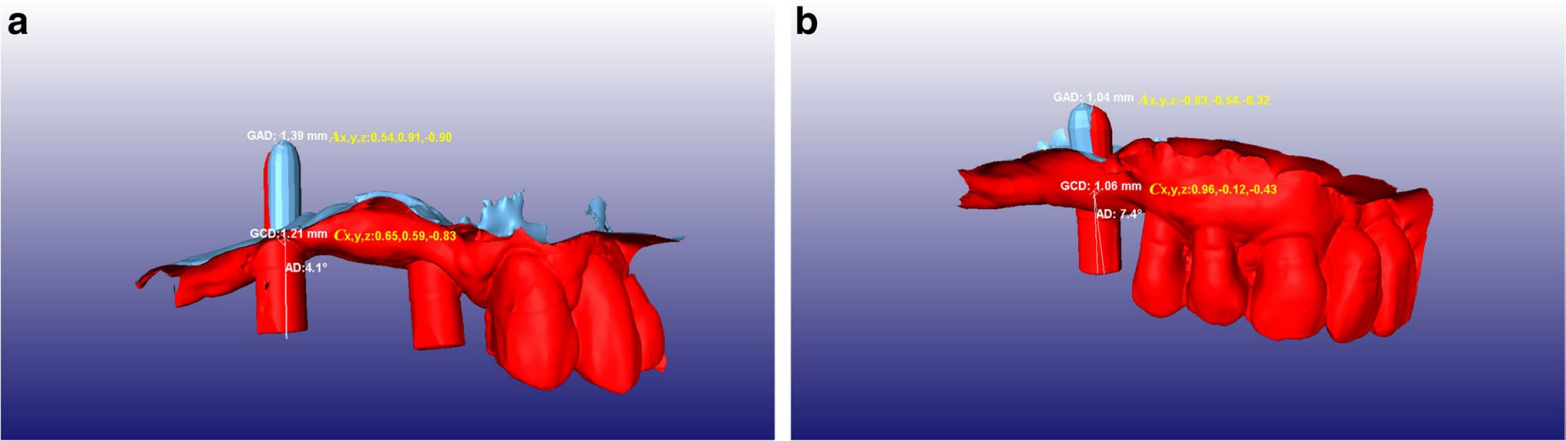
Accuracy analysis. **a** Machine-driven group (patient no. 26). **b** Manual group (patient no. 2). The position of the inserted implant defined by the scan abutment (red), superimposed on the planned position (blue) extracted from the digital plan. GCD, global coronal deviation; *Cx*, *Cy*, *Cz*, vectoral components of GCD; GAD, global apical deviation; *Ax*, *Ay*, *Az*, vectoral components of GAD; AD, angular deviation

The primary outcome variables were angular deviation (AD; the angle closed by the principal axis of the planned implant and the principal axis of the inserted implant in degrees), global coronal deviation (GCD; the distance between the coronal endpoints of the planned and the inserted implants in millimeters), and global apical deviation (GAD; the distance between the apical endpoints of the planned and the inserted implants in millimeters). GCD and GAD were each broken down to vectors in the three-dimensional space (*Cx*, *Cy*, *Cz*, and *Ax*, *Ay*, *Az*, respectively). As for the axes of the coordinate system, *x* marked the mesio-distal dimension, *y* the oro-vestibular dimension, and *z* the cranio-caudal dimension (Fig. 3).

Horizontal coronal deviation (HCD) and horizontal apical deviation (HAD) were calculated from the measured horizontal deviations as a vector sum of *Cx* + *Cy* and *Ax* + *Ay*, respectively. Vertical deviation was measured apically and was equal to *Az*.

The statistical analyses were carried out in SPSS 23.0 (IBM, USA). The measured values were descriptively characterized as means and standard deviations. For the hypothesis tests (between-group comparisons), one-way ANOVA was used. Differences were considered significant at *p* < 0.05.

## Results

The results are summarized in Table 1. Twenty patients were allocated to each study group and each patient received one implant; altogether, 40 implants were inserted in 40 patients. In both groups, distribution of edentulous sites was as follows: 3 single-tooth gaps (15%), 2 multiple-teeth gaps (10%), and 15 free-end tooth gaps (75%).

**Table 1:** Descriptive statistics of the studied parameters in the two study groups and significance levels from the hypothesis tests. *GCD*, global coronal deviation; *Cx*, *Cy*, *Cz*, vectoral components of GCD; *HCD*, horizontal coronal deviation; *GAD*, global apical deviation; *Ax*, *Ay*, *Az*, vectoral components of GAD; *HAD*, horizontal apical deviation; *AD*, angular deviation; *τ*, torque; *t*, time. *Az = vertical deviation (VD)

	Machine-driven group (*N* = 20)		Manual group (*N* = 20)		Intergroup comparison
	Mean	SD	Mean	SD	Significance (*p*)
GCD (mm)	1.20	0.46	1.13	0.31	0.58
*Cx* (mm)	0.81	0.31	0.75	0.32	0.31
*Cy* (mm)	1.00	0.58	0.45	0.39	0.26
*Cz* (mm)	0.44	0.26	0.55	0.25	0.87
HCD (mm)	1.06	0.52	0.92	0.4	0.37
GAD (mm)	1.45	0.79	1.18	0.28	0.17
*Ax* (mm)	0.92	0.39	0.72	0.33	0.70
*Ay* (mm)	0.76	0.88	0.58	0.31	0.72
*Az* (mm)*	0.55	0.28	0.62	0.21	0.52
HAD (mm)	1.28	0.83	0.99	0.28	0.14
AD (°)	4.82	2.07	4.11	1.63	0.23
*τ* (Ncm)	21.75	9.75	18.75	7.05	0.27
*t* (s)	9.25	1.86	36.4	8.15	< 0.001

Mean GCD in the machine-driven group was 1.20 ± 0.46 mm, compared to 1.13 ± 0.38 mm in the manual group. Mean GAD in the machine-driven group was 1.45 ± 0.79 mm, compared to 1.18 ± 0.28 mm in the manual group. HCD averaged 1.06 ± 0.52 mm in the machine-driven group and 0.92 ± 0.40 mm in the manual group. HAD averaged 1.28 ± 0.83 mm in the machine-driven group and 0.99 ± 0.28 mm in the manual group. VD averaged 0.55 ± 0.28 mm in the machine-driven group compared to 0.62 ± 0.21 mm in the manual group. AD averaged 4.82 ± 2.07° in the machine-driven group and 4.11 ± 1.63° in the manual group. Mean insertion torque was 21.75 ± 9.75 Ncm in the machine-driven group, compared to 18.75 ± 7.05 Ncm in the manual group. The mean duration of implant insertion was 9.25 ± 1.86 s in the machine-driven group, compared to 36.40 ± 8.15 s in the manual group.

Statistical analysis did not reveal significant difference between the groups in any of the examined parameters, except for the duration of implant insertion (*F* = 201.84, df = 1, *p* < 0.001), indicating the advantage of the machine-driven approach. *F* and df indicate the value of the *F* statistic and degrees of freedom, respectively.

## Discussion

The present study is the first to investigate if the method of implant insertion (manual or machine-driven) makes a significant difference in the three-dimensional accuracy of implant placement in a half-guided surgical protocol. While equally capable for implant insertion, clinically, both insertion approaches have certain drawbacks. Optimal positioning of the ratchet during insertion usually requires the clinician to use both hands. As a result, reflecting the flap and visualization of the osteotomy site may be challenging. Furthermore, the vertical space required to attach the ratchet is higher compared to machine insertion. On the other hand, the weight of the contra-angled handpiece, the surgical motor, and the attached cables is higher, which might complicate appropriate positioning. Additionally, the procedure is operated with a foot pedal, which may detract attention from the clinician. Despite these characteristic differences, in the present study, we did not find any significant differences between the study groups in any of the accuracy variables.

Based on the lack of differences, it can be argued that the data are homogeneous in terms of accuracy. If so, the averaged accuracy results for the entire studied patient population (without grouping) should be comparable to published results on guided implant surgery in general. Thus, for the purposes of this discussion, we calculated accuracy for the entire population and used these values for comparison with the literature (Table 2). Emphasis was put on global (apical and coronal) deviation, horizontal deviation, vertical deviation, and angular deviation, these being the most frequently reported parameters.

**Table 2:** Descriptive statistics of the accuracy parameters used for comparison with the literature, for the entire patient population (*N* = 40). *GCD*, global coronal deviation; *HCD*, horizontal coronal deviation; *GAD*, global apical deviation; *Az*, vertical deviation; *HAD*, horizontal apical deviation; *AD*, angular deviation

	Mean	SD
GCD (mm)	1.16	0.38
GAD (mm)	1.32	0.54
HCD(mm)	0.99	0.46
HAD (mm)	1.14	0.55
*Az* (mm)	0.59	0.24
AD (°)	4.46	1.85

Kühl et al. in 2013 presented a cadaver study; in 5 lower jaws altogether, 38 implants were placed with flapless guided surgery utilizing half-guided or fully guided protocol. Different dentitions were observed in the lower jaws; thus, both tooth-supported and mucosa-supported guides were used. They found a mean 1.56-mm global coronal deviation, a mean 1.84-mm global apical deviation, and a 4.2° angular deviation in case of half-guided implant placement. They found a non-significant difference in accuracy comparison between the fully guided and half-guided group, and results were comparable to our outcomes [15]. On the other hand, Jung et al. reported in their systematic review that implant position accuracy was better in studies with models and cadavers compared to clinical studies. They postulated that this can be explained by better visual control of the osteotomy axis, a more stable surgical stent position, and no saliva or blood in the models. According to their recommendations, accuracy of guided implant placement should be assessed in clinical situations [16]. Moreover, implant placement accuracy might depend on bone quality and anatomical region. A lower bone density or buccal/lingual undercuts can result in a lower implant accuracy with greater angular deviation after guided surgery [17]. In the present study, the choice of previously sinus-augmented areas in the posterior maxilla was made to avoid heterogeneity of bone morphology and quality of surgical sites.

Global implant position deviations are frequently reported in literature, but only limited data are available on the accuracy of half-guided protocols, only a few of these utilized tooth-supported stents. Ersoy et al. in 2008 reported global coronal deviations of 1.1 ± 0.6 mm after fully guided implant placement using tooth-supported surgical guides. The mean angular deviation was 4.4 ± 1.6° [18]. Di Giacomo et al. in 2012 performed flapless half-guided implant surgeries in edentulous patients and they found a mean of 1.35 ± 0.65-mm global coronal deviation and 1.79 ± 1.01-mm global apical deviation [19]. Our results turned out to be somewhat more favorable, with 1.16 ± 0.38-mm global coronal deviation and 1.32 ± 0.54-mm global apical deviation. Derksen et al. in 2019 reported on even higher accuracy (0.75 ± 0.34-mm global coronal deviation, 1.06-mm ± 0.44-mm global apical deviation), nevertheless in a fully guided clinical setting [20].

The mean angular deviation was 6.53 ± 4.31° in the study of Di Giacomo et al. compared to 4.46 ± 1.85° in the present study [19]. Vercruyssen and co-workers, in 2014, used variously supported surgical guides for the treatment of full edentulism. In their half-guided group, where the surgical guide was mucosa-supported, they found a mean global coronal deviation of 1.23 ± 0.60 mm and a mean global apical deviation of 1.57 ± 0.71 mm [21]. These results are comparable to our findings, even if they indicate slightly less accurate implant placement. In contrast, the mean angular deviation was only 2.86 ± 1.6° in this study, which is lower than what we have found. In general, the results of the present study indicate slightly higher accuracy than those of Di Giacomo et al. and Vercruyssen et al., which is well in line with the observation that tooth-supported guides tend to be slightly more accurate than mucosa- or mucosa and pin–supported guides [22].

Valente and co-workers measured horizontal deviation after half-guided implant placement with various guide support. In their study, the average horizontal deviation between planned and actual implant positions at the coronal and apical ends of the implants were 1.4 ± 1.3 mm and 1.6 ± 1.2 mm, respectively, while the mean angular deviation was 7.9 ± 4.7° [9]. Cassetta et al. in 2012 performed flapless half-guided implant placement with a tooth-supported surgical guide. Fifteen implants were placed in 2 patients. The horizontal apical deviation was 1.28 ± 0.50 mm, and the angular deviation was 4.88 ± 3.38° [23]. These results are very similar to our findings, although in our study implant placement was performed with flap elevation. Our mean horizontal coronal and apical deviations were 0.99 ± 0.46 mm and 1.14 ± 0.55 mm, respectively, and our mean angular deviation was 4.46 ± 1.85°. That is, our study yielded more favorable outcomes in all horizontal parameters than what is reported in the literature.

In our study, we also evaluated the vertical deviation of the placed implant from its planned position. Vertical deviation was measured apically, as the optimal positioning of the implant apex is crucial to avoid interference with adjacent anatomical landmarks (e.g., nasal floor, sinus floor, mandibular nerve canal). Only a few articles reported data on vertical deviation. In the retrospective study by Cassetta et al., the authors inserted 15 implants with tooth support without depth control, and they found that the results were more favorable compared to mucosally supported stents. The mean vertical deviation in these cases was 1.51 ± 1.06 mm [23]. Van de Wiele et al., using mucosa-supported guides, found a mean vertical deviation of 0.75 ± 0.65 mm [12]. In a previously mentioned study, vertical coronal implant deviation was also presented. In this study, implant placement was half-guided but various guide supports were applied. They found a mean of 1.1 ± 1.0-mm apical deviation [9]. Bover-Ramos and colleagues (2018) examined the question of vertical deviation in a systematic review. The analysis was based on six selected clinical studies and found a mean vertical deviation of 0.74 ± 0.10 mm [24]. The mean 0.59 ± 0.24-mm apical vertical deviation we found indicates that we have managed to achieve slightly superior vertical accuracy compared to other studies.

Planned and actual implant positions can be superimposed and compared digitally to characterize outcome accuracy. Comparative studies mainly used postoperative CBCT scans for that purpose. However, in postoperative CBCT scans, implants may cause artifacts due to beam scattering, which is a potential source of measurement error. Furthermore, following the ALARA principles a second CBCT scan should be avoided if the sole purpose is to determine the accuracy of the outcome [25, 26]. The application of intraoral scanners to provide input for such comparisons offers a solution to this problem. The information that an intraoral scan contains on the spatial position of some superstructure (e.g., a scan abutment) attached directly to the implant in the bone makes it possible to reconstruct the spatial position of the implant in the bone, provided that the implant dimensions are known. Application of intraoral scans to detect actual implant location may represent an alternative approach at the same time lowering the total dosage required from the comparison.

By the applied method of comparison, it was possible to avoid additional CBCT scans, whereby we could minimize patient exposure to radiation. The accuracy of CBCT scans was previously reported to be within 0.5 and 0.7 mm but voxel size can influence the final characteristics of the image [27]. In 2017, Renne and co-workers found an average of 79.8 ± 5.17-μm precision and a mean of 48.4-μm trueness using Planmeca PlanScan for sextant scanning, which is one of the best in its category [28]. The presented CBCT followed by intraoral scanning method yielded comparable outcomes to the usual CBCT followed by CBCT alignment.

No significant difference was found between the two study groups in any insertion torque. The four times faster implant placement duration in the machine-driven group was possibly observed due to 50-rpm insertion speed versus the manufacturer’s recommended speed of 15 rpm. The benefit of using a contra-angled handpiece was the effortless acceleration of implant placement compared to the ratchet, which is limited by the operator’s dexterity.

## Conclusion

Within the limits of this study, it can be concluded that half-guided implant placement can result in a favorable implant positioning using a surgical motor, or a torque wrench. Between the two groups, there were no significant differences in terms of accuracy, while implant placement with a surgical motor at a speed of 50 rpm resulted in significantly lower duration. Investigation of implant placement accuracy can be performed based on a preoperative CBCT scan and a postoperative digital intraoral scan, minimizing irradiation dose by avoiding a second CBCT scan.
